# The serum vaspin levels are reduced in Japanese chronic hemodialysis patients

**DOI:** 10.1186/1471-2369-13-163

**Published:** 2012-12-03

**Authors:** Junko Inoue, Jun Wada, Sanae Teshigawara, Kazuyuki Hida, Atsuko Nakatsuka, Yuji Takatori, Shoichirou Kojo, Shigeru Akagi, Kazushi Nakao, Nobuyuki Miyatake, John F McDonald, Hirofumi Makino

**Affiliations:** 1 Department of Medicine and Clinical Science, Okayama University Graduate School of Medicine, Dentistry and Pharmaceutical Sciences, Okayama 700-8558, Japan; 2 Department of Hygiene, Faculty of Medicine, Kagawa University, Kagawa 761-0793, Japan; 3 Millipore, Linco Research, St. Charles, MO, 63304, USA

**Keywords:** Adipokine, End-stage renal disease, Hemodialysis

## Abstract

**Background:**

Visceral adipose tissue-derived serine proteinase inhibitor (vaspin) is an adipokine identified in genetically obese rats that correlates with insulin resistance and obesity in humans. Recently, we found that 7% of the Japanese population with the minor allele sequence (A) of rs77060950 exhibit higher levels of serum vaspin. We therefore evaluated the serum vaspin levels in Japanese chronic hemodialysis patients.

**Methods:**

Healthy Japanese control volunteers (control; n = 95, 49.9±6.91 years) and Japanese patients undergoing hemodialysis therapy (HD; n = 138, 51.4±10.5 years) were enrolled in this study, and serum samples were subjected to the human vaspin RIA system.

**Results:**

The measurement of the serum vaspin levels demonstrated that a fraction of control subjects (n = 5) and HD patients (n = 11) exhibited much higher levels (> 10 ng/ml; Vaspin_High_ group), while the rest of the population exhibited lower levels (< 3 ng/ml; Vaspin_Low_ group). By comparing the patients in the Vaspin_Low_ group, the serum vaspin levels were found to be significantly higher in the control subjects (0.87±0.24 ng/ml) than in the HD patients (0.32±0.15 ng/ml) (p < 0.0001). In the stepwise regression analyses, the serum creatinine and triglyceride levels were found to be independently and significantly associated with the vaspin concentrations in all subjects.

**Conclusions:**

The creatinine levels are negatively correlated with the serum vaspin levels and were significantly reduced in the Japanese HD patients in the Vaspin_Low_ group.

## Background

Visceral adipose tissue-derived serine proteinase inhibitor (vaspin) was identified in the visceral adipose tissue of OLETF (Otsuka Long-Evans Tokushima Fatty) rats, which are used in an animal model of obesity and type 2 diabetes [1]. The mRNA expression of vaspin increases at the peak of obesity and insulin resistance in OLETF rats. The injection of recombinant human vaspin into diet-induced obese mice ameliorates insulin resistance and may be a compensatory factor in the status of obesity [2]. In obese adults [3-6] and children [6,7], the serum vaspin levels increase according to the degree of obesity and insulin resistance and decrease in association with weight reduction brought about by life-style modifications or bypass surgery. The serum vaspin levels in the patients with type 2 diabetes have been demonstrated to be higher [8,9] or similar to [10] those observed in subjects with normal glucose tolerance. Since short-term continuous subcutaneous insulin infusion decreases the plasma vaspin levels associated with improvements in insulin sensitivity [11], the serum vaspin levels may be influenced by both therapeutic modalities and the status of intrinsic insulin secretion in patients with type 2 diabetes.

Some reports have evaluated the vaspin levels in patients with vascular complications. Low vaspin serum concentrations have been found to be correlated with recently experienced ischemic events in patients with carotid stenosis [12]. The presence of microvascular complications is also associated with low vaspin levels [13]. Among patients receiving chronic dialysis therapy, Seeger et al. reported that the mean serum vaspin concentrations are not significantly different between chronic hemodialysis (HD) patients and control subjects, although the vaspin concentrations are positively associated with the serum creatinine levels in HD patients [14]. Recently, we found that 7% of the Japanese population with the minor allele sequence (A) of rs77060950 exhibit higher levels of serum vaspin (10 ng/ml) [15] and that several SNPs within the vaspin gene are significantly associated with the serum vaspin concentrations, reaching P-values of up to 10^-35^ in European populations [16].

Only a small amount of information is available regarding the vaspin levels in HD patients. Serum vaspin levels should be increased in HD patients due to the fact that its small protein size, 50 kDa, which would be freely filtered by the kidneys. Furthermore, vaspin seems to increase with increasing insulin resistance and expanding fat mass. Therefore, it was interesting to know whether serum vaspin levels are elevated or reduced in uremic patients undergoing dialysis, who are frequently in the status of malnutrition. We conducted a cross-sectional clinical study to evaluate whether the serum vaspin levels are altered by the renal function and whether the frequency of much higher levels of serum vaspin is different in HD patients. The ELISA system with a measurement range up to 1 ng/ml (Adipogen) was used in the above mentioned clinical studies. In this study, we employed the RIA system with a wider measurement range from 0.04 ng/ml to 60 ng/ml (Millipore, Linco Research). We measured the serum vaspin levels in healthy Japanese control subjects with a normal renal function and HD patients and compared various clinical parameters.

## Methods

### Patients

Healthy Japanese control subjects (n = 95, 49.9±6.91 years) and patients undergoing hemodialysis therapy (n = 138, 51.4±10.5 years) were enrolled in this study. The healthy control subjects received annual medical health checkups for common disease screening at the Okayama Southern Institute of Health. The control subjects were without any history of medications for diabetes, hypertension and/or dyslipidemia. The patients were receiving chronic hemodialysis therapy (HD) at Shigei Medical Research Hospital, Okayama, Japan. The exclusion criteria were as follows: malignancies, active infectious diseases, hospitalization during the previous 3 months, and initiation of hemodialysis therapy within 6 months. The patients underwent regular hemodialysis three times a week for 4 to 5 hours per session using polysulfone membranes with a bicarbonate-buffered dialysate. The blood flow was maintained in the range of 150-200 ml/min with a dialysate flow of 500 ml/min. The study protocol was approved by the ethics committee of Okayama Southern Institute of Health, Okayama University Hospital and Shigei Medical Research Hospital, and written informed consent was obtained from all participants.

### Biochemical assays and other measurements

Body mass index (BMI) was calculated as the weight in kilograms divided by the square of the height in meters. Waist circumference was measured midway between the lower rib margin and the iliac crest. Blood samples were obtained immediately prior to starting a dialysis session. Laboratory data, including the serum levels of creatinine, plasma glucose, total cholesterol, high-density lipoprotein (HDL) cholesterol, triglycerides (TG), albumin and hemoglobin were measured using an automatic analyzer (Hitachi 7150; Hitachi, Tokyo, Japan). Blood pressure (BP) was measured in the supine position before each dialysis session.

The RIA system, which utilizes ^125^I-labeled vaspin as a tracer and a rabbit polyclonal antibody raised against purified rhvaspin, was used to measure the human vaspin levels in sera (Millipore, Linco Research, St. Charles, MO, USA). Hundred μl of each standard or each sample was pipetted in duplicate into tubes and mixed with 100 μl of human vaspin antibodies. After incubation for 22-24 hours at 4°C, 100 μl of ^125^I-Vaspin tracer was added to all tubes. After incubation for another 22-24 hours at 4°C, 10 μl of rabbit carrier and 1.0 ml of cold (4°C) precipitating reagent were added and incubated for 20 minutes at 4°C. After centrifugation for 20 minutes at 4°C at 2000-3000 x *g*, the supernatant was decanted and the radioactivity of the pellets was counted. Working aliquots of human vaspin standards measuring between 0.01 ng/ml and 60 ng/ml were prepared by performing serial 1:2 dilutions of the stock standard of 60 ng/ml. The log values of the standards were plotted versus the sample-bound counts/total binding counts (B/B_0_) to construct a standard curve to determine the concentrations of serum vaspin in the unknown samples. The linearity of the standard curve was ascertained to be between 0.26 ng/ml and 60 ng/ml. The intra- and inter-assay coefficients of variation (CV) were 5.5% and 12.3% at 4.37 ng/ml and 2.7% and 12.4% at 8.44 ng/ml human vaspin concentrations, respectively. The spike recovery and linearity were in a range of 95.3-101.4% and 100.0-116.2%, respectively.

### Statistical methods

The descriptive data are presented as the mean ± SD. The statistical analyses were performed by a Windows personal computer with statistics software JMP 7 (SAS Institute Inc., Cary, NC, USA). *P*-values of <0.05 or <0.01 were considered to be statistically significant. Differences between two groups were assessed by using the Mann–Whitney U test. Correlations between the vaspin levels and clinical characteristics were evaluated with the Spearman’s rank correlation test. Stepwise regression analyses based on backward elimination were performed to test the influence of various factors on the vaspin serum concentrations.

## Results

### The serum vaspin levels in the control and HD patients

Measurement of the vaspin levels in the human serum samples revealed that the majority of subjects displayed vaspin levels between 0.042 and 1.57 ng/ml. A minor fraction of subjects (n = 5 in the control group and n = 11 in the HD group) displayed much higher levels, ranging from 16 to 46 ng/ml. Therefore, we classified the participants into two subgroups based on the vaspin serum levels: Vaspin_Low_: vaspin levels < 3 ng/ml and Vaspin_High_: vaspin levels > 10 ng/ml. Henceforth, all of the statistical analyses in the present study were performed among subgroup Vaspin_Low_, unless otherwise indicated (Table 1). The serum vaspin levels were 0.54±0.33 ng/ml (range: 0.042-1.57 ng/ml) in the total samples and were significantly higher in the control subjects (0.87±0.24 ng/ml) than in the HD patients (0.32±0.15 ng/ml) (p < 0.0001). The serum vaspin levels remained lower in the HD patients than in the control subjects following adjustment for the hemoglobin levels (0.32±0.15 ng/ml *v.s.* 0.92±0.32 ng/ml, p < 0.0001) and BMI (0.32±0.15 ng/ml *v.s.* 0.85±0.22 ng/ml, p < 0.0001).

**Table 1:** **Baseline characteristics of the healthy controls with lower serum vaspin levels (control/Vaspin**_**Low**_**) or higher serum vaspin levels (control/Vaspin**_**High**_**) and the chronic hemodialysis (HD) patients with lower serum vaspin levels (HD/Vaspin**_**Low**_**) or higher serum vaspin levels (HD/Vaspin**_**High**_**)**

	**Control/Vaspin**_**Low**_	**HD/Vaspin**_**Low**_	***P *values**	**Control/Vaspin**_**High**_	**HD/Vaspin**_**High**_	***P *values**
Total number	90	127		5	11	
Vaspin (ng/ml)	0.87 ± 0.24	0.32 ± 0.15	<0.0001*	23.4 ± 6.26	31.0 ± 9.15	0.0785
Gender (female: male)	48:42	41:86	0.0019*	4:1	5:6	0.0023*
Age (years)	49.7 ± 6.90	51.9 ± 10.1	0.0575	52.8 ± 5,47	45.4 ± 13.7	0.1788
BMI (kg/m^2^)	23.5 ± 3.84	22.0 ± 3.46	0.0115*	22.6 ± 1.62	24.3 ± 4.79	0.3948
Waist circumference (cm)	78.1 ± 11.5	77.8 ± 9.69	0.8701	74.2 ± 7.32	78.5 ± 9.91	0.3596
Systolic BP (mmHg)	124 ± 16	132 ± 24	0.0049*	135 ± 16	128 ± 27	0.5561
Creatinine (mg/dl)	0.73 ± 0.15	13.2 ± 2.91	<0.0001*	0.74 ± 0.21	13.0 ± 3.00	<0.0001*
Blood glucose (mg/dl)	98.6 ± 28.0	119.2 ± 43.8	<0.0001*	87.6 ± 9.48	115.0 ± 33.0	0.0250*
Total cholesterol (mg/dl)	214.4 ± 36.0	162.2 ± 40.1	<0.0001*	211.2 ± 17.0	166.9 ± 37.8	0.0080*
HDL cholesterol (mg/dl)	66.4 ± 15.1	45.9 ± 13.9	<0.0001*	65.4 ± 14.8	52.0 ± 16.7	0.1467
TG (mg/dl)	135.0 ± 86.1	127.2 ± 93.1	0.5397	85.2 ± 24.4	97.2 ± 27.9	0.4147
Albumin (g/dl)	4.58 ± 0.24	3.85 ± 0.32	<0.0001*	4.56 ± 0.15	3.65 ± 0.35	<0.0001*
Hemoglobin (g/dl)	14.0 ± 1.64	10.3 ± 0.89	<0.0001*	12.4 ± 2.78	10.1 ± 0.69	0.1361

The data are presented as the number, ratio or mean ± s.d. *, p < 0.05.

*Vaspin* Visceral adipose-tissue derived serpin, *BMI* Body mass index, *SFA* Subcutaneous fat area, *VFA* Visceral fat area, *BP* Blood pressure, *HDL* High-density lipoprotein, *TG* Triglycerides.

In the HD patients, there were no significant differences in the serum vaspin concentrations between females (0.35±0.13 ng/ml) and males (0.30±0.16 ng/ml) as well as between diabetic (0.30±0.11 ng/ml) and nondiabetic (0.32±0.16 ng/ml) patients. Similarly, in the control subjects, the circulating vaspin levels were not significantly different in the gender groups (females: 0.89±0.21 vs. males: 0.85±0.27 ng/ml).

### Univariate correlations

In the analysis of the total participants, including the control and HD patients, the serum vaspin levels were found to be statistically correlated with BMI and the levels of plasma glucose, total cholesterol, HDL-cholesterol, triglycerides, albumin and hemoglobin. In the control group, the vaspin levels were significantly correlated with the triglyceride levels. However, the serum levels of vaspin did not show any statistical correlations with any of the parameters in the HD patients (Table 2).

**Table 2:** **Univariate analyses of the healthy controls (n = 90), chronic dialysis patients (HD; n = 127) and all subjects, including both the healthy controls and chronic dialysis patients (n = 217), with lower serum vaspin levels (Vaspin**_**Low**_**) using the circulating vaspin levels as dependent variables**

	**Control/Vaspin**_**Low**_		**HD/Vaspin**_**Low**_		**All subjects**	
	***r***	***P *values**	***r***	***P *values**	***r***	***P *values**
Age	0.0331	0.7663	-0.0827	0.3556	-0.1046	0.1309
BMI	0.1184	0.2866	0.0191	0.8638	0.2182	0.0047*
Waist circumference	0.0810	0.4667	0.0209	0.8154	0.0563	0.4171
Creatinine	-0.0192	0.8630	0.1526	0.0881	-0.7464	<0.0001*
Blood glucose	0.1772	0.1090	-0.0218	0.8096	-0.1764	0.0108*
Total cholesterol	0.1697	0.1250	0.0987	0.2962	0.5282	<0.0001*
HDL cholesterol	-0.1901	0.0852	0.0043	0.9634	0.4107	<0.0001*
TG	0.3430	0.0015*	0.1212	0.1991	0.1838	0.0097*
Albumin	-0.0149	0.8934	0.0741	0.4117	0.6462	<0.0001*
Hemoglobin	0.0066	0.9526	0.0352	0.6944	0.6784	<0.0001*

*, p < 0.05. *Vaspin *Visceral adipose-tissue derived serpin, *BMI* Body mass index, *HDL *High-density lipoprotein, *TG *Triglycerides.

### Multivariate correlations

Multiple regression analyses were performed to test whether any of the following factors influence the serum vaspin concentrations: the creatinine and triglyceride levels. When correcting for these variables using stepwise regression analyses, the levels of creatinine and triglycerides were found to be independently and significantly associated with the vaspin concentrations in all subjects (Table 3). The levels of triglycerides significantly predicted the serum vaspin levels in the control group. In the HD patients, no significant correlations were detected between the vaspin levels and any parameter.

**Table 3:** **Multivariate linear regression analyses of the healthy controls (n = 90) and all subjects, including both the healthy controls and chronic hemodialysis patients (n = 217), with lower serum vaspin levels (Vaspin**_**Low**_**) using the circulating vaspin levels as dependent variables**

**Models**	**Independent variables**	**β**	**Standardized β**	**βSE**	**t value**	**P value**	**Model r**^**2**^
1	All subjects						
	Age	-0.003	0.002	-0.070	-1.247	0.214	0.586
	BMI	0.001	0.005	0.006	0.104	0.917	
	Hemoglobin	0.012	0.014	0.077	.829	0.409	
	TG	0.001	0.000	.0154	2.643	0.009*	
	Albumin	0.148	0.070	0.063	0.692	0.490	
	Creatinine	-0.030	0.005	-0.620	-6.511	0.000*	
	Blood glucose	0.000	0.001	-0.030	-0.511	0.610	
2	Control						
	Age	-0.001	0.004	-0.035	-0.311	0.757	0.119
	BMI	-0.001	0.007	-0.010	-0.084	0.933	
	TG	0.001	0.000	0.353	-3.016	0.003*	

*, p < 0.05. *Vaspin* Visceral adipose-tissue derived serpin, *BMI* Body mass index, *TG* triglycerides.

## Discussion

Obesity has undoubtedly influenced the growth of end-stage renal disease (ESRD) worldwide [17]; however, some studies have suggested that higher BMIs are associated with improved survival in patients with ESRD [18,19]. Adipokines derived from adipose tissues regulate energy homeostasis, insulin sensitivity, lipid metabolism, coagulation, fibrinolysis, inflammation and vascular functions [20]. Therefore, measuring the levels of adipokines in patients with ESRD may be beneficial for predicting cardiovascular events and patient survival. In patients with chronic kidney disease (CKD), the levels of adipokines appear to increase in association with declines in the glomerular filtration rate (GFR). This is most likely due to reduced renal metabolism of adipokines, which may increase vascular risks in patients with a reduced renal function [20]. Among the adipokines, leptin and adiponectin are well-established vasoactive substances, and the serum levels of both leptin and adiponectin have been investigated in patients with ESRD. In patients with incidental coronary heart disease (CHD), the leptin levels exhibit a moderate association with incident CHD [21]. In dialysis patients, poor appetite is associated with fat mass loss and declining leptin levels [22]. Similarly, Scholze et al. demonstrated that low leptin levels predict poor outcomes in dialysis patients, which most likely reflects the detrimental effects of protein energy wasting and loss of fat mass on outcomes in this population [23]. Regarding adiponectin, earlier reports have suggested that higher adiponectin levels are linked to better outcomes [24,25]; however, recent studies show contradictory results [26,27]. Drechsler et al. reported that increased longitudinal levels of adiponectin are associated with higher risks of adverse cardiovascular outcomes and death and that this association is weakened by the confounding effects of increased levels of N-terminal prohormone brain natriuretic peptide (NT-pro-BNP)[26]. These studies suggest that the adipokines levels in patients with ESRD are influenced by the renal function, nutritional state and status of inflammation and may affect the vascular function and clinical outcomes.

In the current study, a fraction of the Japanese population, including both healthy subjects and HD patients, exhibited 10-fold higher levels of serum vaspin of more than 10 ng/ml, as demonstrated using the human vaspin RIA system with wider concentration ranges of measurement. Repeated measurement of the serum vaspin levels in this population (Vaspin_High_) always demonstrated similarly high levels of vaspin, and one of the subjects’ parents also exhibited similar levels. Therefore, such higher vaspin levels are primarily defined by genetic factors. Indeed, we demonstrated that a significant subpopulation (7%) of both the subjects with normal fasting glucose levels (n = 259) and patients with type 2 diabetes (n = 275) displayed much higher levels (10-40 ng/ml; Vaspin_High_ group), while the serum vaspin levels of 93% of the samples varied from 0.2 to 3 ng/ml in the Vaspin_Low_ group. According to genotyping, rs77060950 is tightly linked to the serum vaspin levels, i.e. CC (0.6±0.4 ng/ml), CA (18.4±9.6 ng/ml) and AA (30.5±5.1 ng/ml) (p < 2 ×10^-16^)[15]. In the current study, we demonstrated that a similar frequency in the Vaspin_High_ group was observed among the HD patients, who seemed to be genetically defined. Furthermore, the population with vaspin levels higher than 10 ng/ml included different ethnic groups, i.e. ~1% in European populations, as noted in previous reports [14]. We also demonstrated that ~1% of the Danish twin population exhibit vaspin levels higher than 10 ng/ml using the human vaspin RIA system [28]. By separating the patients in the Vaspin_High_ and Vaspin_Low_ groups, we clearly demonstrated that the serum vaspin levels were significantly reduced in the HD patients in the Vaspin_Low_ group. Seeger et al. did not demonstrate differences in the mean serum vaspin concentrations between chronic hemodialysis (HD) patients and controls, which may be due to the fact that their analysis combined both Vaspin_High_ and Vaspin_Low_ groups together. Since the serum vaspin levels correlate with BMI, another explanation for the lower vaspin levels observed in Japanese HD patients may be related to the fact that lower BMI levels are observed in Japanese HD patients than in German HD patients [14].

Lower serum vaspin levels in HD patients are a unique feature, since most adipokines, including leptin, adiponectin and resistin, are higher in ESRD and HD patients. Since the molecular mass of vaspin is ~50 kDa [1], vaspin may not be efficiently eliminated by hemodialysis. The presence of lower serum vaspin levels in HD patients suggest the existence of extra renal elimination of vaspin from the circulation, including the function of clearance receptors on the cell surface of various cells and tissues. Since the serum vaspin levels are well-correlated with BMI and the levels of albumin and hemoglobin, vaspin may reflect the nutritional status of HD patients. Recently, the circadian rhythm of the serum vaspin levels has been reported [29]. The serum vaspin levels are highest in the early morning before breakfast and fall to the trough levels within two hours after breakfast. The serum vaspin levels also exhibit preprandial rises and postprandial falls at lunch and dinner, although to lesser degrees than that observed at breakfast [30]. In addition, central and peripheral vaspin administration acutely reduces food intake and exerts sustained blood glucose-lowering effects in mice [30], suggesting the possible role of vaspin in appetite control. The relationship between the reduced vaspin levels observed in HD patients and appetite loss was not determined in the current study, and future studies are required to demonstrate a such link. Since vaspin may exert insulin-sensitizing effects and exhibit anti-inflammatory properties in the obesity state [1,2], reduced vaspin levels in HD patients may be linked to adverse cardiovascular events. The current study is a cross-sectional clinical study and has some limitations. Cohort studies are required to demonstrate whether lower serum vaspin levels are related to poor outcomes in HD patients. To demonstrate such relationships in a cohort study, genetically defined Vaspin_High_ and Vaspin_Low_ groups should be analyzed separately and the Vaspin_High_ group should be excluded from using vaspin as a marker. Separation of the Vaspin_High_ and Vaspin_Low_ groups is also important for comparing different ethnic groups, and future studies are required to demonstrate whether the current findings can also be applied to other ethnic populations.

## Conclusion

In summary, measuring the serum vaspin levels demonstrated that ~7% of both healthy controls and HD patients exhibit much higher levels (> 10 ng/ml; Vaspin_High_ group), while the rest of the population exhibits lower levels (< 3 ng/ml; Vaspin_Low_ group). By comparing the patients in the Vaspin_Low_ group, the serum vaspin levels were found to be significantly higher in the control subjects (0.87±0.24 ng/ml) than in the HD patients (0.32±0.15 ng/ml) (p < 0.0001). In the stepwise regression analyses, the levels of creatinine and triglycerides were found to be independently and significantly associated with the vaspin concentrations in all subjects. Future studies are required to determine whether vaspin is a marker of eating behavior and nutrition in HD patients and to demonstrate the importance of the vaspin levels for predicting the outcomes of HD patients.

## Competing interests

The authors declare that they have no competing interests.

## Authors’ contributions

JI, JW, YT, SK, SA, KN and HM participated in the design of the study and recruitment of the patients. JI, ST and JFM established the RIA assay system and obtained all of the measurements. JW, KH and AN participated in the cloning of the vaspin gene and production of recombinant vaspin proteins. JI, JW and HM conceived of the study, participated in coordination and performed the statistical analyses. All authors have read and approved the final manuscript.

## Pre-publication history

The pre-publication history for this paper can be accessed here:

http://www.biomedcentral.com/1471-2369/13/163/prepub
